# Zn-Based Three-Dimensional Metal-Organic Framework for Selective Fluorescence Detection in Zwitterionic Ions

**DOI:** 10.3390/ijms26083566

**Published:** 2025-04-10

**Authors:** Hongbin Liu, Yue Zhao, Biyi Huang, Hui Liu, Putao Zhang, Wen Gu, Tingli Ma

**Affiliations:** 1 Nanxun Innovation Institute, Zhejiang University of Water Resources and Electric Power, Hangzhou 310018, China; 2 School of Electrical Engineering, Zhejiang University of Water Resources and Electric Power, Hangzhou 310018, China; 3 College of Materials and Chemistry, China Jiliang University, Hangzhou 310018, China; 4 School of Future Technology, Henan University, Kaifeng 475001, China; 5 Key Laboratory of Advanced Energy Materials Chemistry (MOE), College of Chemistry, Nankai University, Tianjin 300071, China; 6 Graduate School of Life Science and Systems Engineering, Kyushu Institute of Technology 2-4 Hibikino, Fukuoka 808-0196, Japan

**Keywords:** zinc-based MOF, fluorescence probe, dual-ion detection, rapid response

## Abstract

Zinc-based MOFs exhibit significant advantages in ion detection due to their unique structure and chemical properties. They can efficiently and selectively recognize and detect specific ions, making them powerful analytical tools for applications in environmental monitoring, biomedical fields, and more. In this work, we used a simple ligand to improve the coordination environment of Zn^2+^ ions and successfully synthesized a 3D coordination compound Zn(all-bdc)(Py) MOF through a straightforward hydrothermal method at low temperature. Additionally, we explored the potential of this MOF as a bifunctional ion fluorescence probe for both cationic and anionic recognition. The results showed that this 3D porous MOF exhibited excellent recognition ability for trivalent iron ions (Fe^3+^) and potassium permanganate (KMnO_4_^−^) ions due to its highly porous structures and efficient ion recognition. When iron ions were added to 500 μL and potassium permanganate ions were added to 100 μL, the fluorescence of the compound was effectively quenched, and the detection limits for these two ions were 0.95 μM and 0.13 μM, respectively. The mixed-ion experiments also demonstrated that even in the presence of similar ions, this 3D MOF still maintained good selective recognition ability, specifically identifying Fe^3+^ and KMnO_4_^−^ ions. This work provides a novel synthetic strategy for the design of MOFs capable of mixed-ion recognition and detection, expanding their application potential in ion sensing and analysis.

## 1. Introduction

Metal-Organic Frameworks (MOFs) are compounds formed by the self-assembly of metal ions or clusters with organic ligands, offering high surface areas and porous structures. Due to their high tunability, ultra-high surface area, and excellent chemical stability, these materials have shown immense potential in areas like gas storage, catalyst design, drug delivery, and environmental remediation [1,2,3]. One of their key advantages is the ability to adsorb and concentrate trace pollutants, improving detection sensitivity and accuracy [4,5]. In atmospheric pollution monitoring, they can capture and concentrate low concentrations of volatile organic compounds or greenhouse gases such as carbon dioxide and methane [6,7]. In aquatic environments, these materials can be designed with specific pore sizes and chemical functionalities to selectively adsorb heavy metal ions or organic pollutants from water [8,9]. Their chemical functionality can be adjusted by altering metal ions, organic ligands, or synthesis conditions, enabling the selective recognition and adsorption of various pollutants. For example, certain types are highly efficient at adsorbing heavy metals such as lead, mercury, and cadmium, reducing their impact on ecosystems and human health [10,11,12]. Additionally, they can be used to remove dyes, pesticides, and other organic pollutants through specific interactions with pollutants, allowing for selective capture and separation [13,14,15]. Overall, their versatility and specificity make them highly effective for environmental monitoring, pollution control, and the development of efficient filtration systems [16,17].

Traditional detection methods typically rely on high-precision instruments with specific functions to perform qualitative or quantitative analyses of target substances. However, these methods are undeniably costly due to the high expense of specialized equipment and materials, and they often have a limited detection range. In contrast, the use of MOFs in detection offers clear advantages [18,19,20]. They are cost-effective, easy to operate, and involve relatively simple physical changes during detection, typically involving energy transfer without altering the framework structure [21,22]. This makes it easier to separate and recycle the materials after detection. Additionally, MOF-based probes have a broad range of applications, including metal ions, small organic molecules, and various pollutants [23,24]. MOFs have shown good performance in detecting these diverse targets [25,26,27]. These benefits have made MOF-based detection a popular and rapidly developing research direction, offering an efficient alternative to traditional detection methods [28,29,30]. Among them, transition metal-based MOFs have the widest range of applications and the best performance [31,32]. Their high compatibility with organic ligands, stable coordination structures, multidimensional frameworks, and precisely sized pores enable efficient capture and enrichment of target ions [33]. Additionally, the organic ligands and metal ions in transition metal-based MOFs can interact specifically with target ions through mechanisms such as intermolecular hydrogen bonding and covalent bonding, allowing for highly selective ion detection [34]. This high sensitivity and selectivity give transition metal-based MOFs significant advantages in trace ion detection [35]. Omer et al. synthesized a novel Mn-based MOF that functions as a cold/hot-adapted and recyclable oxidase nanozyme (Km 0.085 mM), further developed for the ratiometric-based, colorimetric, and color tonality visual-mode detection of nitrite in water and food [36]. Similarly, Zhang et al. successfully synthesized a series of ratiometric fluorescent probes based on Zr-based MOFs using a one-pot method. These dual-responsive RhB-doped Zr-based MOF probes, attributed to fluorescence resonance energy transfer (FRET), photoinduced electron transfer (PET), and competitive absorption mechanisms, effectively detect Cu^2+^ and Fe^3+^ ions [37]. Bifunctional MOF-based fluorescent probes have attracted considerable attention due to their ability to detect both anions and cations [38]. These multifunctional probes significantly improve detection efficiency, making them particularly suitable for complex systems where multiple ions coexist, such as biological fluids and environmental water samples. However, most of the highly effective bifunctional fluorescent probes reported to date are based on lanthanide metal MOFs [39,40]. Fluorescent probes based on transition metal MOFs with detection limits below 1.0 μM are still rare [41]. Therefore, the development of efficient bifunctional fluorescent probes based on transition metal MOFs remains highly necessary.

In this study, we synthesized a Zn-based MOF by using a simple cost-effective method, used it as a cation–cation dual-ion detector to selectively detect Fe^3+^ and KMnO_4_^−^ in water, and achieved good results. After adding a solution containing ferric ions or potassium permanganate ions, the fluorescence intensity of the Zn(all-bdc)(Py) MOF was significantly weakened. Based on our calculations, the detection limits for Fe^3+^ and MnO_4_^−^ ions were both below 1.0 μM, specifically 0.95 μM and 0.13 μM, respectively, which means they are at the forefront of those reported in the literature [41]. At the same time, we also tested the ability of Zn(all-bdc)(Py) to selectively detect dual ions by mixing with other types of cations or anions. The results also show that in the presence of other cations and anions, the MOF probe we synthesized can still play a good role and can selectively detect Fe^3+^ and KMnO_4_^−^. This has a good reference role in the identification of ferric ions and potassium permanganate ions in the fields of medicine, the environment, materials, etc.

## 2. Results and Discussion

**Figure 1:**
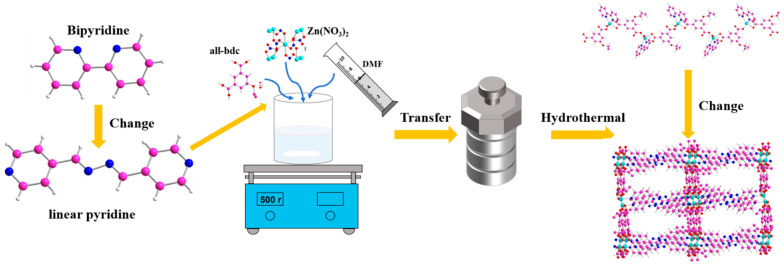
Schematic diagram of the construction of three-dimensional Zn-based MOF.

By adjusting the ligands involved in coordination, the structure and porosity of MOFs can be well adjusted, which is a major feature of MOF design and synthesis. Therefore, when we replaced bipyridine with a smaller coordination angle with straight-chain pyridine with a larger coordination angle, we successfully adjusted the spatial structure of Zn-based MOF from one dimension to three dimensions. The strategy is shown in Figure 1.

**Figure 2:**
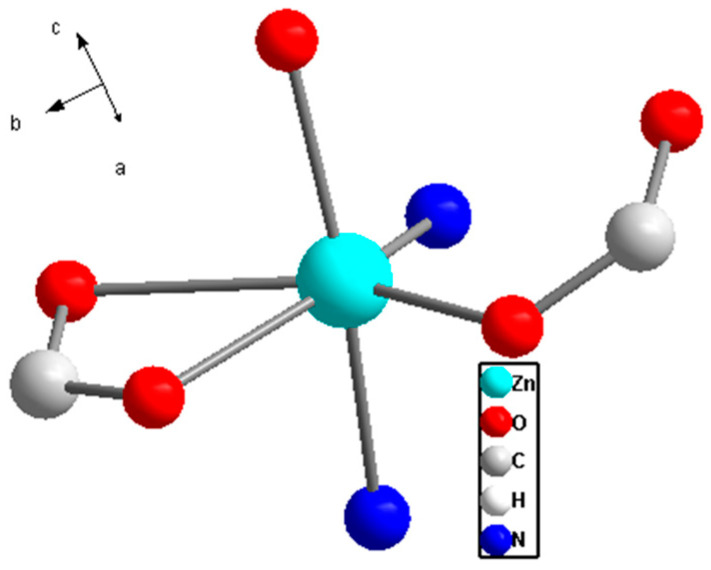
Coordination environment of the Zn^II^ in complex Zn(all-bdc)(Py).

The results from crystal structure analysis indicate that this compound belongs to the monoclinic crystal system with the P21/c space group. The overall structure exhibits C_2h_ symmetry, featuring a 2-fold symmetry axis and a horizontal mirror plane (Figure 2). From the crystallographic perspective, the Zn^II^ ion in the basic unit of this Zn-based MOF adopts a distorted octahedral coordination geometry with six coordinating atoms. Four of these coordinating atoms are oxygen atoms from the carboxyl groups of three different all-bdc ligands. One ligand contributes both oxygen atoms from its carboxyl group, while the other two carboxyl groups each contribute one oxygen atom. The remaining two coordination sites are occupied by nitrogen atoms from two different linear pyridine (Py) molecules.

**Figure 3:**
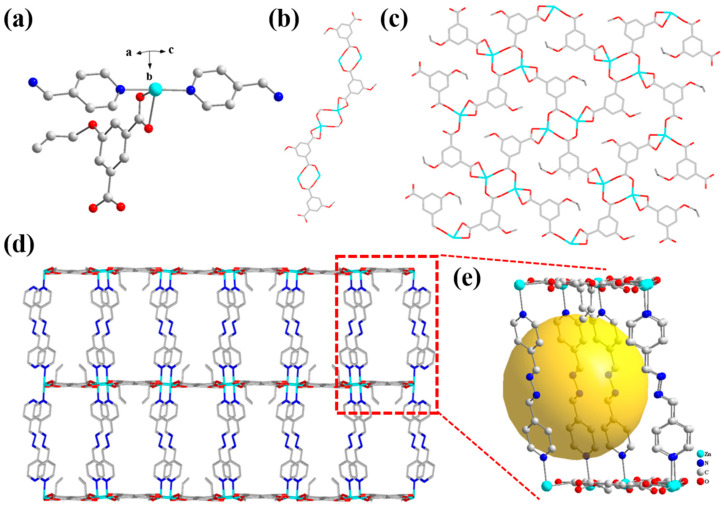
(**a**) The asymmetric structure of the Zn-based MOF. (**b**) View of 1D chain (only all-bdc is considered). (**c**) The 2D layer structure of the Zn-based MOF. (**d**) The 3D structure of the Zn-based MOF viewed along different views and (**e**) its porous structure.

Thus, the minimum asymmetric unit of the Zn(all-bdc)(Py) complex consists of one Zn²⁺ ion, one deprotonated all-bdc ligand, and one linear Py molecule (Figure 3a). The Zn-O (All-bdc) bond lengths range from 2.027 to 2.351 Å, while the Zn-N (Py) bond lengths range from 2.140 to 2.163 Å. In terms of spatial configuration, by replacing the original 1,4-bipyridine with linear pyridine, we overcame the issue of close coordination sites, which typically leads to a structure with excessive coordination to a single metal ion and insufficient additional coordination sites for metal–metal interactions. The all-bdc ligand connects with Zn^2+^ ions to receive a one-dimensional chain and connects with other all-bdc ligands to obtain a 2D layer (Figure 3b,c). This adjustment resulted in the formation of a three-dimensional layered structure for the synthesized Zn(all-bdc)(Py) complex, where the layers are connected by linear pyridine molecules (Figure 3d). This design resolves the problem of the one-dimensional chain structure that would have been observed if 1, 4-bipyridine were used. Meanwhile, the designed three-dimensional MOF features a porous structure with pore sizes of approximately 1.2 nm (Figure 3e). This porous framework facilitates the free diffusion of target ions, providing favorable conditions for interaction.

**Figure 4:**
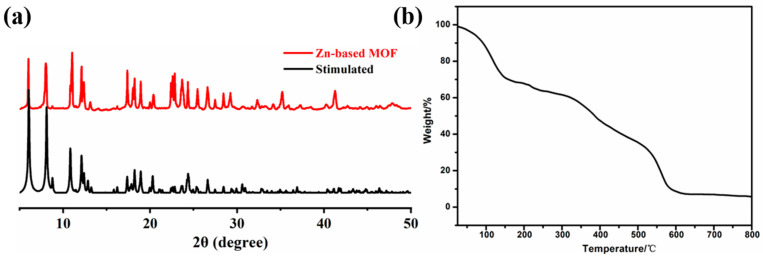
(**a**) XRD patterns and (**b**) thermogravimetric analyses of the Zn-based MOF.

X-ray diffraction (XRD) was used to analyze the purity of the complex, and the results are presented in Figure 4a. By comparing the experimental pattern with the simulated pattern, we observed that the characteristic peaks align well with those of the simulated curve. Therefore, it can be concluded that the synthesized Zn-based MOF is predominantly pure-phase. In the practical application of MOF materials, the stability determines whether the material can maintain long-term performance and be recycled. Therefore, in order to test the thermal stability of the Zn(all-bdc)(Py) complex, thermogravimetric analysis (TGA) was used, and the results are shown in Figure 4b. The Zn-based MOF was placed in a thermal analyzer, and the weight loss of the material was tested from 25 °C to 800 °C, so as to obtain the thermal stability of the complex. From the figure, we can see that the first weight loss process of Zn(all-bdc)(Py) MOF is before 154 °C, and the weight loss is about 29.14%, which is the loss of solvent DMF molecules. The second process occurs between 154 °C and 500 °C, which is the collapse process of Zn-based MOF. Starting from 500 °C, the collapse begins to accelerate. At 600 °C, the collapse is complete, with a weight loss of 90.2%, and the remaining 9.8%, which is zinc oxide, and the theoretical value is 12.1%. These results indicate that the synthesized Zn-based MOF maintains structural stability at room temperature and even under relatively high temperatures, supporting its long-term performance stability and potential for sustainable reuse.

**Figure 5:**
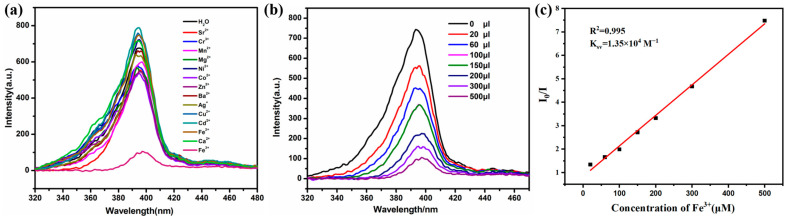
(**a**) Comparison of the luminescence intensity of the Zn(all-bdc)(Py) MOF upon adding 500 μL of different metal ions. (**b**) The luminescence intensity of the Zn(all-bdc)(Py) MOF in the presence of various contents of Fe^3+^. (**c**) Stern–Volmer plots of Fe^3+^ ion. I is the luminescence intensity with Fe^3+^, and I_0_ is the luminescence intensity without Fe^3+^.

When the Zn-MOF is photoexcited, its internal electrons transition from the ground state to the excited state and then generate fluorescence during the de-excitation process [42]. Therefore, using Zn-based MOFs as fluorescent probes to detect target substances through fluorescence quenching is one of their key applications [43,44]. Based on this principle, we conducted ion sensitivity tests on the Zn(all-bdc)(Py) MOF. As a pre-designed zwitterionic probe, we first examined its recognition of cations. A 4.0 mg sample of the ground complex was placed in a vial, and 5.0 mL of distilled water was added, followed by 30 min of ultrasonication to ensure thorough dispersion. During the experiment, metal nitrate solutions (1.0 × 10^−2^ mol/L) were gradually added separately, with cations including Sr^2+^, Cr^3+^, Mn^2+^, Ni^2+^, Co^2+^, Zn^2+^, Ba^2+^, Ag^+^, Fe^3+^, K^+^, Cu^2+^, Cd^2+^, Na^+^, Fe^2+^, and Ca^2+^. From the results, after adding a certain concentration of iron ions to the solution of the Zn-based MOF, the fluorescence intensity of the complex decreased significantly (Figure 5a). As Fe^3+^ was incrementally added to the Zn(all-bdc)(Py) MOF solution, the fluorescence intensity gradually weakened. When the added volume reached 500 μL, the fluorescence was completely quenched (Figure 5b). This indicates that the Zn-based MOF exhibits good selectivity toward Fe^3+^. As shown in Figure 5c, there is a good linear correlation between the quenching efficiency of the Zn-based MOF and the amount of Fe^3+^ in a concentration range of 0–500 µM (R^2^ = 0.995). The detection limit was calculated to be 0.95 µM based on 3σ/k (σ: standard error; k: slope), which indicates that the MOF fluorescent probe we synthesized can be used as a potential detection agent for detecting Fe^3+^ in aqueous solution. The comparison with other fluorescent probes is shown in Table 1, which also proves the superior level of detection of our probe.

**Figure 6:**
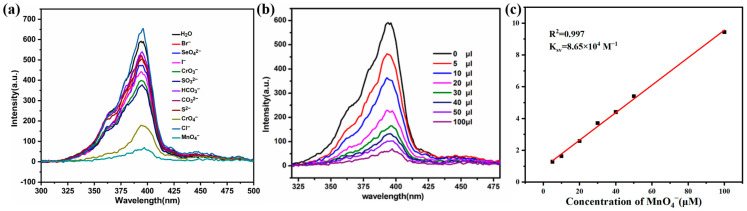
(**a**) Comparison of the luminescence intensity of the Zn(all-bdc)(Py) MOF upon adding 100 μL of different anions. (**b**) The luminescence intensity of the Zn(all-bdc)(Py) MOF in the presence of various contents of MnO_4_^−^. (**c**) Stern–Volmer plots of MnO_4_^−^ ion. I is the luminescence intensity with MnO_4_^−^ ion, and I_0_ is the luminescence intensity without MnO_4_^−^ ion.

At the same time, we conducted an anion sensitivity test for the Zn(all-bdc)(Py) MOF. In the experiment, 4.0 mg of Zn(all-bdc)(Py) MOF was ground and placed in a vial, followed by the addition of 5.0 mL of distilled water. The mixture was sonicated for 30 min to ensure thorough dispersion. Gradually, sodium salt solutions of different anions (1.0 × 10^−2^ mol/L) were separately added to the Zn-based MOF aqueous solution. The tested anions included Br^−^, SeO_4_^2−^, I^−^, CrO_3_^−^, SO_3_^2−^, HCO_3_^−^, CO_3_^2−^, S^2−^, CrO_4_^2−^, Cl^−^, and MnO_4_^−^. The experimental results indicate that when a certain concentration of permanganate ions (MnO_4_^−^) was introduced into the Zn-based MOF solution, the fluorescence intensity of the MOF significantly decreased (Figure 6a). As MnO_4_^−^ solution was gradually added, its fluorescence intensity progressively weakened. When the added amount reached 100 μL, a clear fluorescence quenching effect was observed (Figure 6b). This demonstrates that the Zn-based MOF exhibits good selectivity in recognizing anion of MnO_4_^−^. As shown in Figure 6c, the quenching efficiency of Zn-based MOF also exhibits a good linear correlation with the MnO4^−^ concentration (R² = 0.997). The calculated detection limit is 0.13 μM, which is lower than that of most reported MOFs. A detailed comparison of detection limits with other reported MOF probes is also provided in Table 1.

**Figure 7:**
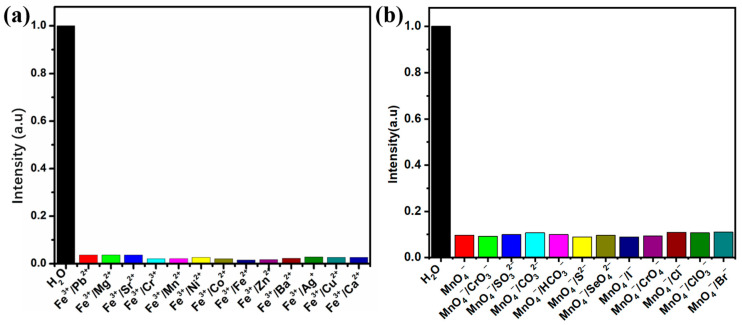
Comparison of the luminescence intensity of the Zn(all-bdc)(Py) MOF immersed in the presence of (**a**) 500ul Fe^3+^ and other metal cations and (**b**) 100 μL MnO_4_^−^ and other anions.

In practical applications, MOF materials used as probes often encounter complex environments, and ion detection must have good anti-interference ability, especially to avoid interference from similar ions, in order to achieve accurate identification and efficient detection. Therefore, we conducted an experiment to evaluate the anti-interference ability of the Zn(all-bdc)(Py) MOF in detecting Fe^3+^. We simultaneously added 500 μL of Fe^3+^ and other metal ions of equal concentration to the complex solution to evaluate the recognition ability of the Zn-based MOF as a fluorescent probe in complex conditions. The experimental results showed that even in the presence of other metal ions, the fluorescence intensity of Zn(all-bdc)(Py) still exhibited significant quenching (Figure 7a), demonstrating its excellent anti-interference performance in Fe^3+^ detection. In addition, we further tested the anti-interference performance of the Zn(all-bdc)(Py) MOF in detecting MnO_4_^−^ anions. Similarly, we added 100 μL of MnO_4_^−^ along with other anions of equal concentration to the MOF solution to investigate its selective recognition ability for MnO_4_^−^. The experimental results revealed that despite the presence of other anions, the fluorescence intensity of the Zn(all-bdc)(Py) MOF still showed a significant decline (Figure 7b), indicating that this fluorescent probe also exhibits excellent anti-interference ability when detecting target anions. In summary, these two anti-interference experiments strongly confirm that the synthesized Zn(all-bdc)(Py) MOF, as a dual-functional fluorescent probe, can effectively recognize specific target ions in complex environments, demonstrating outstanding selectivity and stability.

**Figure 8:**
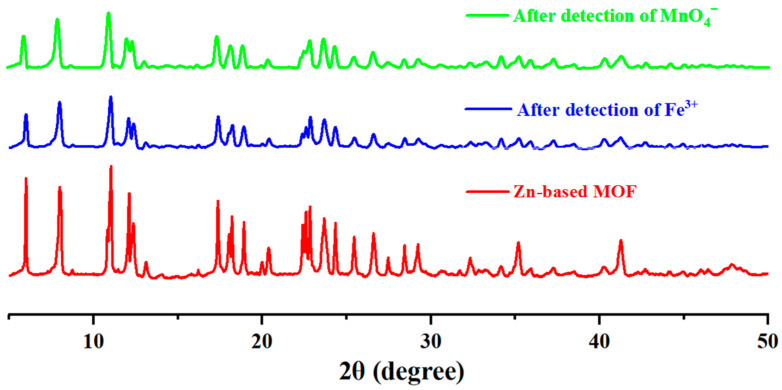
XRD patterns of Zn-based MOF before and after ion detection.

For a fluorescent probe to be reusable, it is essential to ensure structural stability before and after experiments. To confirm that the structure of the Zn-based MOF remained unchanged, we performed XRD analysis on the samples after detection. As shown in Figure 8, after detecting Fe^3+^ and MnO_4_^−^ ions and subsequent separation, the main peaks of the sample exhibited no significant changes. This demonstrates that the synthesized MOF maintains excellent structural stability.

For the fluorescence quenching mechanism of our synthesized Zn-based MOF toward Fe^3+^ and MnO_4_^−^, we analyzed it in conjunction with previous studies [49,53]. First, the XRD pattern of the Zn-based MOF after Fe^3+^ detection showed no significant changes in the main peaks compared to the original pattern, indicating that the fluorescence quenching was not caused by structural collapse or ion exchange. Therefore, the interaction between Fe^3+^ ions and the organic ligands of the Zn-based MOF is likely the primary reason for fluorescence quenching. Additionally, the uncoordinated nitrogen atoms from the linear pyridine moieties provide open Lewis base sites, which facilitate binding with external metal ions, leading to an enhanced quenching effect. Similarly, the XRD pattern of the Zn-based MOF after MnO_4_^−^ detection also showed no significant changes in the main peaks, suggesting that the fluorescence quenching was not due to structural collapse or ion exchange. Instead, the host–guest interaction is likely the dominant factor for fluorescence quenching [54]. This is most likely due to the strong absorption of MnO_4_^−^ in the 350–500 nm range, which overlaps with the excitation wavelength of Zn-MOF, thereby leading to fluorescence quenching [50].

**Figure 9:**
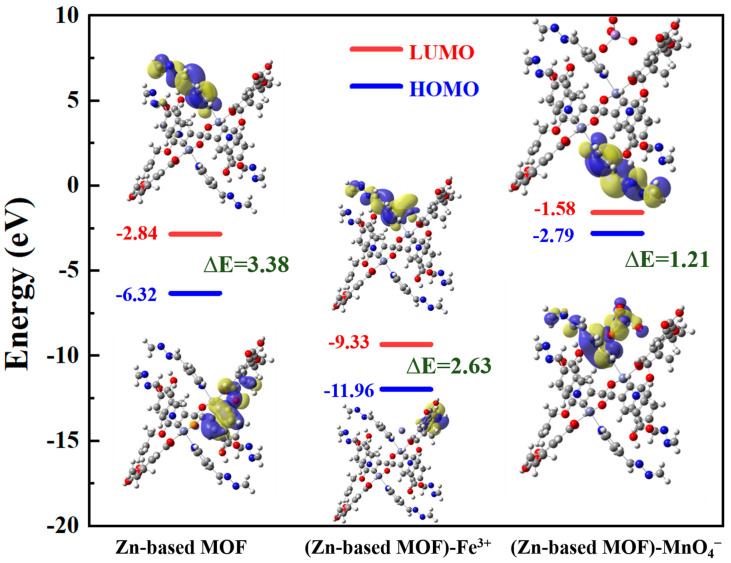
LUMO and HOMO orbitals of Zn-based MOF, (Zn-based MOF)-Fe^3+^, and (Zn-based MOF)- MnO_4_^−^.

To further confirm the fluorescence quenching mechanism of Zn-based MOF detection, we performed DFT theoretical calculations, and the results are shown in Figure 9. For the Zn-based MOF monomer, the HOMO electron density is mainly distributed on the carboxyl oxygen and pyridine rings of the ligand molecule, while the LUMO electron density is located on the pyridine rings adjacent to the metal coordination sites [55]. After forming the (Zn-based MOF)-Fe^3+^, the HOMO shifts to the all-bdc ligand, while the LUMO is primarily located on Fe^3+^ and the pyridine rings of the ligand. These results indicate that Fe^3+^ inhibits charge transfer in the excited state of the Zn-based MOF, ultimately leading to fluorescence quenching. The Zn-based MOF exhibits a strong absorption band at 395 nm, corresponding to the HOMO-LUMO bandgap transition with ΔE = 3.38 eV. Meanwhile, the (Zn-based MOF)-Fe^3+^ has a lower energy level (ΔE = 2.683 eV), indicating that the (Zn-based MOF)-Fe^3+^ structure is more stable [56]. Therefore, the interaction between the Zn-based MOF probe and Fe^3+^ can be attributed to the coordination of Fe^3+^ with the uncoordinated nitrogen sites, forming a relatively stable state that prevents intramolecular charge transfer, ultimately causing fluorescence quenching. For the (Zn-based MOF)-MnO_4_^−^, the HOMO and LUMO electron density distributions remain largely unchanged, while the bandgap is significantly smaller (ΔE = 1.21 eV). This suggests that MnO_4_^−^ absorbs energy in the same spectral range as the MOF excitation, leading to competitive energy absorption and fluorescence quenching of the Zn-based MOF.

Our results demonstrate that Zn-based MOFs have significant potential as fluorescent probes for ion detection. With continuous advancements in synthesis techniques and the emergence of novel Zn-based MOFs, the sensitivity and accuracy of these materials in ion detection are expected to improve further. By optimizing pore structures, introducing more efficient functional groups, and developing new detection strategies, Zn-based MOFs will be capable of detecting ions at even lower concentrations with high precision. Moreover, by tuning the structure and functional groups of Zn-based MOFs, selective detection of different types of ions can be achieved, thereby expanding their application range. As detection technologies continue to evolve, Zn-based MOFs are also expected to enable the simultaneous detection of multiple ions, greatly enhancing detection efficiency and meeting the demands of complex systems. Despite their promising prospects, Zn-based MOFs still face several challenges. For instance, improving their stability and recyclability remains crucial for extending their lifespan and reducing detection costs. Additionally, optimizing the synthesis process to enable large-scale production while maintaining cost-effectiveness is another key challenge. To address these issues, several strategies can be considered. Further research should focus on enhancing the stability and recyclability of Zn-based MOFs by exploring new methods and technologies. Refining synthesis conditions can also improve production efficiency and reduce costs. Furthermore, interdisciplinary collaborations between chemistry, materials science, and environmental science will be essential in driving the development of Zn-based MOFs for ion detection applications.

## 3. Materials and Methods

### 3.1. Materials

All the chemicals were of analytical grade and used as received without further purification. Zinc nitrate (≥99%), 1, 4-bis(4-pyridyl)-2, 3-diaza-1, 3-butadiene (Py) (≥99%), N, N-Dimethylformamide (DMF) (≥99%), ethanol (≥99%), dimethylacetamide (≥99%), dimethyl5-hydroxyisophthalate (≥99%), potassium carbonate (≥99%), allyl bromide (≥99%), anhydrous sodium sulfate (≥99%), and sodium hydroxide (≥99%) were purchased from Sigma-Aldrich Co. LLC., St. Louis, MO, USA.

### 3.2. Measurements

FT-IR spectra were obtained by using a Bio-Rad FTS6000 spectrophotometer, and the wavelength range was from 4000 to 400 cm^−1^ (KBr pellets). Powder Xray diffraction (PXRD) was performed on a Rigaku D/max-IIIA diffractometer (CuKa, λ = 1.54056 Å). Thermogravimetric analysis (TGA) curves were obtained by using a NETZSCH TG 209 analyzer, and the temperature range was from 25 to 800 °C under nitrogen flow with a heating rate of 10 °C min^−1^. As for the theoretical calculation methods, structural optimization and orbitals were performed using DFT/Lanl2dz with the quantum chemical package Gaussian 09.

### 3.3. X-Ray Structure Determination and Structure Refinement

Single-crystal X-ray diffraction data for the Zn-based MOF were collected with a Bruker SMART APEX CCD instrument with graphite monochromatic Mo Ka radiation (λ = 0.71073 Å). Absorption corrections were made by multi-scan methods. The structures were solved with the program Olex2-1.5 and refined by full matrix least squares methods on all F2 data with Shelxtl-2019. The SQUEEZE subroutine of the PLATON-241123 software suite was applied to remove the scattering from highly disordered solvent molecules. All fluorescence measurements were performed on an MPF-4 fluorescence spectrofluorometer.

### 3.4. Synthesis of Ligand of All-bdc

The ligand of 5-(allyloxy)isophthalic acid (C_11_H_10_O_5_) (all-bdc) was synthesized according to a previous study [57]. We dissolved 1.015 g (0.0406 mol) of 5-propyleneoxyisophthalic acid dimethyl in 50.0 mL of 95% ethanol aqueous solution. Then, we mixed this solution with 50 mL of an aqueous solution containing 0.649 g (0.162 mol) of NaOH. Once the mixture was a solution, it was reacted at 65 °C for 15 h. After the reaction solution was cooled to room temperature, the ethanol was removed by rotary evaporation at 40 °C in a water bath. Then, 2.0 mol·L^−^¹ hydrochloric acid solution was added to adjust the pH to 6.5, ultimately yielding a white solid product with a yield of 78.7%. NMR data analysis: (300 MHz, CDCl_3_): δ = 8.26 (s, 1 H); 7.75 (s, 2 H); 6.02 (ddt, ^3^J(H-3, H-1) = 17.4 Hz, ^3^J(H-3, H-2) = 10.4 Hz, ^3^J(H-3, H-4) = 5.2 Hz, 1 H, H-3); 5.36 (dd, ^3^J(H-1, H-3) = 17.4 Hz,^3^J(H-2, H-3) = 10.4 Hz, 2 H, H-1, H-2); 4.61 (d, ^3^J(H-4, H-3) = 5.2 Hz, 2 H, H-4); 3.92 (s, 6 H).

### 3.5. Synthesis of Zn-Based MOF

A total of 0.0226 g (0.10 mmol) all-bdc, 0.0200 g (0.10 mmol) Py, and 0.0297 g (0.10 mmol) Zn(NO_3_)_2_·6H_2_O were mixed with 10.0 mL DMF and stirred for 30 min until the solution was clear. The mixture was stirred for 30 min until the solution became clear. The solution was transferred to a 25 mL PTFE reaction vessel with a stainless-steel protective outer jacket, sonicated for 30 min to ensure thorough mixing, and then placed in a blast drying oven. The program was set to heat the mixture to 80 °C for 1.5 h, maintaining the temperature at 80 °C for 96 h, followed by cooling to room temperature over 48 h. After the reaction, the product was filtered and washed with distilled water. FT-IR (KBr pellets, cm^−1^): 3431m, 3076w, 2935w, 2349w, 1684s, 1604s, 1453s, 1382s, 1249s, 1143m, 1099s, 1037s, 930s, 779s, 726s, 691s, 531s.

Yellow cubic transparent crystals were selected from the product and fixed on a single-crystal diffraction apparatus. Diffraction data were collected at a temperature of 113 K. Intensity correction was performed using the SADABS-8.40B and SAINT-2016 software packages. The crystallographic data of the Zn-MOF are stored in the Cambridge Crystallographic Data Centre. The CCDC number of the Zn-MOF is 2427350. The crystallographic data are reported in Table 2.

## 4. Conclusions

We used a dicarboxylic acid ligand combined with a long-chain pyridine to improve the coordination environment of zinc metal ions, successfully synthesizing a three-dimensional structure of the complex Zn(all-bdc)(Py) MOF. The thermal stability of the Zn-based MOF was significantly improved after the coordination environment was improved. At the same time, the practical effect of this MOF synthesized by a simple hydrothermal method as a novel type of zwitterionic fluorescence detection probe has been well verified. It can detect potassium permanganate ions as an anion detection probe and trivalent iron ions as a cation detection probe. After adding the target ions, the fluorescence intensity of the aqueous solution of the Zn(all-bdc)(Py) MOF changed significantly. When 500 μL cation of Fe^3+^ and 100 μL anion of MnO_4_^−^ were added, the fluorescence of the complex almost disappeared, indicating quenching. Through calculations, the detection limits for these two ions were determined as 0.95 μM and 0.13 μM. At the same time, we also conducted ion sensitivity experiments on the detection of the complex. The results showed that the complex could selectively detect Fe^3+^ in the presence of other cations and selectively detect MnO_4_^−^ in the presence of other anions. This demonstrated that the Zn-based MOF, as a bifunctional fluorescent probe, can effectively identify specific target ions in complex environments and show good selectivity and stability. This provides valuable reference for the application of MOFs used as probes in the selective detection of specific ions in complex environments.

## Figures and Tables

**Table 1:** Performance comparison between various MOF fluorescent probes.

MOF Fluorescent Probe	Detection Limits (μM)		Ref.
	Fe^3+^	MnO_4_^−^	
Zn-based MOF	4.33	–	[45]
Cd-based MOF	4.01	–	[46]
Cd-based MOF	3.56	–	[47]
Eu-based MOF	3.13	–	[48]
Zn-based MOF	0.18	–	[49]
Zn-based MOF	–	4	[50]
Y-based MOF	2.38	1.29	[41]
Zn-based MOF	–	0.24	[51]
Eu-based MOF	–	1.36	[52]
Tb-based MOF	–	0.0448	[40]
Zn-based MOF	0.95	0.13	This Work

**Table 2:** Crystallographic data of Zn-based MOF.

Complex	Zn(All-bdc)(Py)
Structural formula	Zn(all-bdc)(Py)
Empirical formula	C22H18N4O5Zn
Formula weight	483.78
Temperature/K	386.3
Crystal system	monoclinic
Space group	P21/c
a/Å	15.564(2)
b/Å	16.3295(16)
c/Å	15.6433(19)
α/°	90.00
β/°	110.266(4)
γ/°	90.00
Volume/Å^3^	3729.6(8)
Z	4
ρcalcg/cm^3^	0.862
μ/mm^−1^	0.683
F(000)	992.0
Crystal size/mm3	0.2 × 0.2 × 0.2
Independent reflections	8486 [Rint = 0.0414, Rsigma = 0.0338]
Data/restraints/parameters	8486/0/298
Goodness-of-fit on F2	1.374
Final R indexes [I ≥ 2σ (I)]	R1 = 0.06820, wR2 = 0.2125
Final R indexes [all data]	R1 = 0.0700, wR2 = 0.2243
Largest diff. peak/hole/e Å−3	2.05/−0.47

## Data Availability

Data are contained within the article.
